# Acute stress reduces population-level metabolic and proteomic variation

**DOI:** 10.1186/s12859-023-05185-4

**Published:** 2023-03-07

**Authors:** Katherine F. Steward, Mohammed Refai, William E. Dyer, Valérie Copié, Jennifer Lachowiec, Brian Bothner

**Affiliations:** 1 Department of Chemistry and Biochemistry, Montana State University, Bozeman, MT 59717 USA; 2 Thermal Biology Institute, Montana State University, Bozeman, USA; 3 Department of Plant Sciences and Plant Pathology, Montana State University, Bozeman, USA

**Keywords:** Proteomics, Metabolomics, Cellular stress, Canalization

## Abstract

**Background:**

Variation in omics data due to intrinsic biological stochasticity is often viewed as a challenging and undesirable feature of complex systems analyses. In fact, numerous statistical methods are utilized to minimize the variation among biological replicates.

**Results:**

We demonstrate that the common statistics relative standard deviation (RSD) and coefficient of variation (CV), which are often used for quality control or part of a larger pipeline in omics analyses, can also be used as a metric of a physiological stress response. Using an approach we term Replicate Variation Analysis (RVA), we demonstrate that acute physiological stress leads to feature-wide canalization of CV profiles of metabolomes and proteomes across biological replicates. Canalization is the repression of variation between replicates, which increases phenotypic similarity. Multiple in-house mass spectrometry omics datasets in addition to publicly available data were analyzed to assess changes in CV profiles in plants, animals, and microorganisms. In addition, proteomics data sets were evaluated utilizing RVA to identify functionality of reduced CV proteins.

**Conclusions:**

RVA provides a foundation for understanding omics level shifts that occur in response to cellular stress. This approach to data analysis helps characterize stress response and recovery, and could be deployed to detect populations under stress, monitor health status, and conduct environmental monitoring.

**Supplementary Information:**

The online version contains supplementary material available at 10.1186/s12859-023-05185-4.

## Background

Cellular stress response (CSR) is mediated through numerous molecular mechanisms to maintain homeostasis. For example, DNA damage repair, the unfolded protein response, mitochondrial stress signaling, and regulated cell death are all global stress pathways [1]. These programs are initiated by a diverse group of signaling molecules that includes metabolites and proteins. Metabolomics and proteomics methods are thus well suited for investigating CSR, as they capture global snapshots of an organism’s cellular state at a given time [2, 3]. This global phenotypic information, built from individual molecules helps explain not only stress, but also disease states, antibiotic or herbicide resistance, and evolutionary fitness [4] by characterizing phenotypic plasticity relative to baseline conditions [2, 5, 6]. Studies investigating CSR generally focus on a specific stressor, model system, or signaling pathway. In this study, we find that acute stress globally decreases molecular variability in plants, animals, and microorganisms and that such measures are useful for understanding CSR.

Standard omics workflows typically report variability among individuals and groups to quantify the reliability of an experiment. Multiple metrics are used including relative standard deviation (RSD) or Coefficient of Variation (CV), hierarchical clustering, principal component analysis (PCA), as well as multivariate statistical analyses [7]. CV is used in omics analyses to evaluate the repeatability of a biological assay or the precision of an experiment [8] and is reported as a ratio of the standard deviation to the mean. Variability in data is generally considered to be undesirable, and many methods have been employed to minimize intra-group variation among biological replicates [9–11]. Nonetheless, intrinsic phenotypic variability among individuals in a population has been exploited to provide population-level insights into the fields of ecology, evolution, and genetics [12]. A recent study demonstrated that while the sigma factor σV lysozyme stress response is heterogenous in a *Bacillus subtilis* population, changing the amount of stress can push the population towards a more homogenous lysozyme resistance, reducing the phenotypic variability [13]. Historically, going back to 1862, Yablokov et al. used standard deviation and CV metrics to report ranges of phenotypic states within a population of marine mammals and proposed that such data informs on how new taxa arise [14]. More recently, it was reported that the CV of metabolites decreased due to acute stress in animals and microorganisms [15].

We now expand this foundation by characterizing and comparing CV profiles of metabolome and proteome data from resting and stress-challenged organisms as a tool to describe CSR. Multiple publicly available and in-house omics datasets were analyzed to assess changes in CV profiles in plants, animals, and microorganisms, in response to acute stress. Changes in CV means and medians were determined, and CV distribution profiles were analyzed [16] to complete what we term Replicate Variation Analysis (RVA). RVA is described in the context of a standard metabolomics workflow, including multivariate clustering and PCA analyses of treatment groups. The outcome of our analyses is a correlation between acute stress and reduced variation in global metabolite and protein profiles. This finding holds for a variety of organisms inclusive of both bacterial and eukaryotic species, including plants.

## Results

**Fig. 1:**
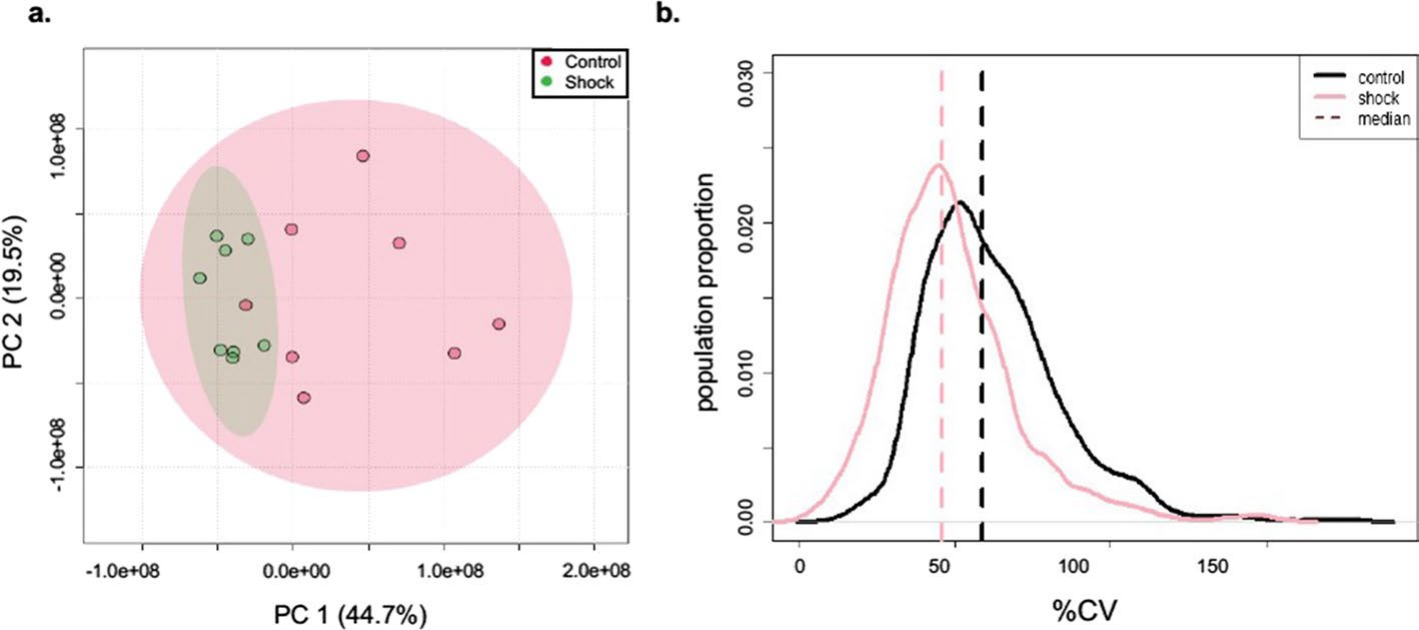
Metabolic variation in response to hemorrhagic shock in a mammal. **A** Principal component analysis of control (red) and shocked (green) *S. scrofa* (n = 8). **B** Profile distribution plots of the CV of metabolite features from *S. scrofa* replicates from a control (black) and a shocked group (pink). The X axis shows the CV and the Y axis is the proportion of metabolites in the metabolome. Adapted from Heinemann et al. (2014)

This project began with our previous observation that CV distributions of metabolomes (n = 8) derived from urine were altered during hemorrhagic shock in *S. scrofa* [17]. The focus of that work was to identify relevant stress biomarkers. Our reanalysis of the data revealed that metabolic variation among individuals was significantly reduced during hemorrhagic shock. A 2D PCA score plot indicated that data variability is canalized under stress with a reduction in both PC1 and PC2 (Fig. 1A). Further analysis revealed that stress is associated with a reduced median CV value (from 59 to 46% and a significantly reduced mean CV (62–49%, Wilcoxon t-test < 0.001), and a CV distribution that is more narrow (Kolomogrov–Smirnov [K–S] test, d = 0.287, *p* < 0.001, Fig. 1B). To establish if decreased variation in metabolite abundance among biological replicates is a general outcome of acute stress, we analyzed another metabolomics project that we had recently published.

**Fig. 2:**
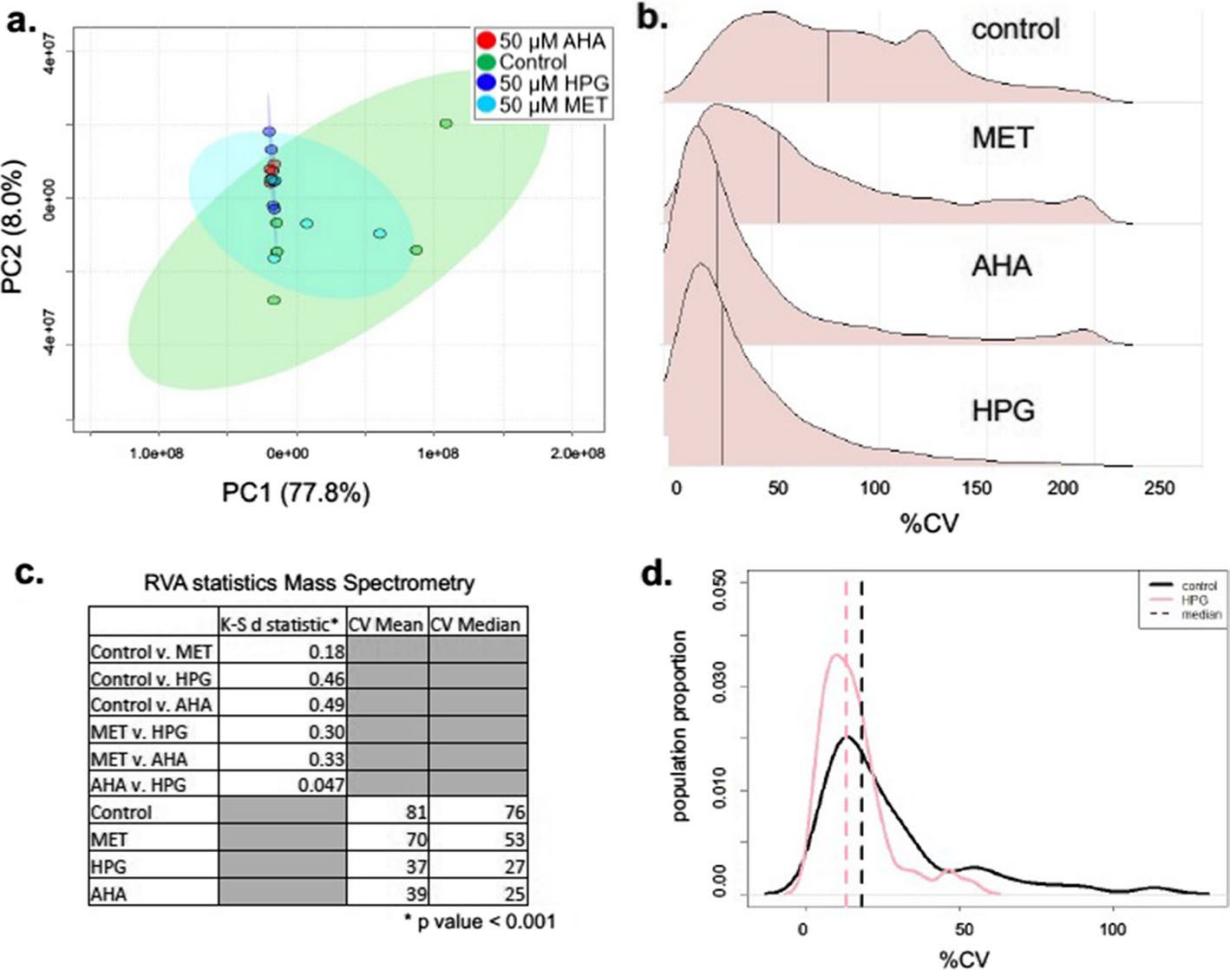
Metabolic variation in *E. coli* treated with non-canonical amino acids. **A** Principal component analysis of four different treatment groups from non-canonical amino acid treatment experiments on *E. coli* cell cultures with median displayed as a solid line (red = AHA treatment, green = control, blue = HPG and cyan = MET) (Steward et al. 2020). **B** Distribution plots of CV of mass spectrometry metabolite feature profiles for the non-canonical amino acid treated cultures of *E. coli*. **C** Table of CV statistics include the K–S d statistic for the different comparisons of the Control to the other groups, the CV mean and the CV median. **D** Profile distribution plots of the CV of NMR metabolite features from *E. coli* replicates from a control (black) and HPG treated (pink)

Our attention turned to a data set that investigated the metabolic impact of stress-inducing Bio Orthogonal Non-Canonical Amino Acid Tags (BONCAT) on the growth of *Escherichia coli* [18]. Batch cultures of *E. coli* were grown on minimal medium (Control) or with additions of methionine (MET), azidohomoalanine (AHA), or homopropargylglycine (HPG) (n = 5). Intracellular metabolite profiles were analyzed using both MS and NMR-based metabolomics techniques. 2D PCA analysis of the MS metabolomics data (Fig. 2A) revealed that the control cultures displayed greater variation among biological replicates than the stress treatment groups. When the same mass spectrometry data were analyzed using RVA, changes in the CV profiles between the control and amino acid tag additions were also observed (Fig. 2B). CV means and medians were decreased, and the distribution profiles narrowed with a sharper peak (Fig. 2C). The NMR data revealed a similar pattern in distribution between the control and HPG samples (K–S test, d = 0.28, *p* = 0.022) with a median decrease from 18% (control) to 13% (HPG) and a significant decrease in mean CV (control = 26%, HPG = 15%, Wilcoxon t-test, *p* = 0.0015; Fig. 2D). The RVA approach demonstrated that metabolomic dysregulation in HPG was greater than AHA, which was greater than MET, and all three treatments caused a decrease in variation relative to the control, a pattern mirrored in the NMR metabolomics data as well (Additional file 1: Fig. S1A). The RVA distribution profiles matched the differential abundance analysis of the original work, in which we showed that the HPG, AHA and MET additions resulted in significant perturbation to 19, 11, and 7% of the metabolites, respectively. RVA thus has the potential to be used as a measure of stress, as it correlates to dysregulation of analytes.

**Fig. 3:**
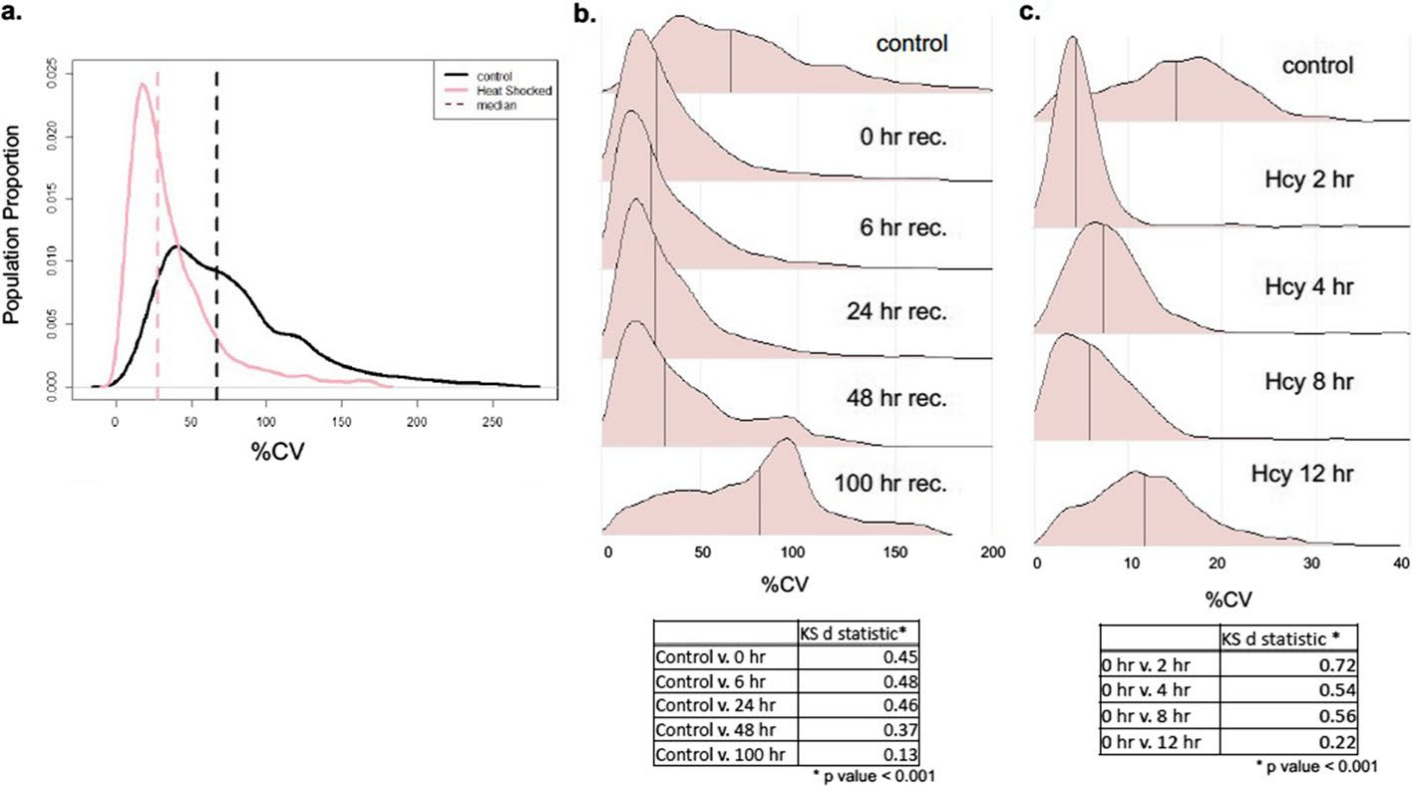
Distribution of CV in *A. fatua* and temporal RVA analysis. **A** Distribution profile plot of metabolomic CV of *A. fatua* exposed to heat shock at 40 C (pink) and the control group (black). **B** Temporal CV profiles from heat stressed *A. fatua*. Time post-stress is from zero to 100 h of recovery. Table below: values of the K–S test. **C** Temporal CV profiles of methionine dependent cancer cell line supplemented with homocysteine (hcy) in the growth media, with timepoints collected after 2, 4, 8 and 12 h of acclimation. Table below: values of the K–S test

We next analyzed data from physiological investigations of the weedy plant *Avena fatua* (wild oat). To examine the global impact of this acute stress, we inflicted a heat shock treatment (40 °C, 24 h) on inbred seedlings, followed by metabolomics analyses after increasing durations of recovery (n = 8). This study demonstrated that CV distribution profiles were markedly altered soon after heat shock (Fig. 3A). Median CV values were reduced from 67% in untreated plants to 28% in heat shocked plants. Mean CV values also showed a significant change between untreated and heat shock groups (control = 76%, heat shock = 37%, Wilcoxon T test *p* < 0.001). As documented for S*. scrofa* and *E. coli* above, CV distributions were also significantly canalized following heat shock (K–S test, d = 0.46, *p* < 0.001).

The *A. fatua* data were also analyzed to assess the kinetics of recovery from stress and how this impacts CV distribution. Throughout the 100-h recovery period, CV distribution means increased from 37 to 76% (Wilcoxon t-test *p* < 0.001) while K–S test d values decreased from 0.45 to 0.13, indicating less difference from the unstressed CV distribution (Fig. 3B). During recovery, the CV distribution widened and became less peaked with the metabolome approaching a distribution that resembled data from untreated plants. As seen in the *E. coli* data above, the temporal CV distribution profiles of heat shock and recovery in *A. fatua* suggest that a qualitative measure of stress can be assessed based on CV distributions of the population.

### Analysis of public omics data sets

To examine the generality of our approach and observations, a series of published data sets from other research groups was analyzed. A structured approach to finding and utilizing data from public repositories was used: we selected studies that involved acute stressors that would result in a stress response or acclimation. Experimental regimes that included significant cell or organism die-off were excluded so that CSR was not complicated by system or pathway shutdown that occurs during death. We then confirmed that post-processed data was supplied, to eliminate potential bias from processing through our in-house pipeline. A significant and unexpected limitation in identifying pertinent datasets was the lack of sufficient metadata and documentation so that data could be assigned to a specific experimental group and the origin of numerical values was clear.

### Metabolomics data

We employed our RVA approach on MS-based metabolomics data that tracked the metabolic adaptations of a methionine sensitive cancer cell line [19]. The original experiment involved replacing methionine in the growth medium with homocysteine, followed by an acclimatization period (n = 4). The cell lines stressed by the loss of methionine failed to thrive in its absence, but supplementation with homocysteine resulted in adaptations that enabled cell growth. RVA analyses of the metabolic mass spectral features demonstrated that the stress imparted by the absence of methionine resulted in significant canalization of CV profiles (K–S test, d = 0.72, *p* < 0.001) (Additional file 1: Fig. S1B). This pattern was also reflected in mean CV values, which decreased from 15 to 6% (Wilcoxon t-test *p* < 0.001) for the control and stressed groups, respectively, and median CV values decreased from 15 to 4%.

This cancer cell dataset was of particular interest because it also included a temporal analysis of CSR. Adaptation to homocysteine was tracked over 12 h by periodic removal of metabolite samples from untreated and methionine-stressed cells. The CV profiles indicated that the peaked profile of early time points shifted to a wider distribution resembling that of the control group, and KS-test d statistic changed from 0.72 to 0.22 between the 2 h and 12 h time points, again reflecting a CV distribution that is trending towards the unstressed control (Fig. 3C).

The second external dataset came from a study in which *Neocloeon triangulifer* (mayfly adults) were fasted overnight and then subjected to heat stress or ambient temperature [20]. Metabolite samples (n = 6) were analyzed by LCMS. RVA analysis revealed subtle changes in CV values, which displayed a slight decrease in the mean from 23 to 20%, and median CV decrease (18.1% to 16.5%) from the ambient temperature insects as compared to the heat shocked group. Although mean and median changes were small, CV distributions tended towards a canalized profile in the heat exposed group (K–S test, d = 0.078, *p* = 0.037) (Additional file 1: Fig. S1C). The difference in CV profiles reflect the impact of acute thermal stress, even under a shared fasting condition.

The next three datasets had acute stress treatments through diet or environmental adjustment, types of stress not previously discussed. The third external data set originated from a metabolomics study that investigated the impact of diet on *Mus muscula* (house mouse) intestinal digesta composition. The treated group was fed a low protein, low fat chow to mimic malnourishment, and mass spectrometry metabolite data were collected from control and diet-restricted mice (n = 4) [21]. RVA analysis demonstrated a clear change in CV distribution profiles (KS-test, d = 0.48, *p* < 0.001), with a change in median CVs from 48 (control) to 21 (diet) and mean CVs (control = 48%, diet = 21%, Wilcoxson’s t-test < 0.001) (Additional file 1: Fig. S2A). The fourth and fifth datasets originated from a study in which *Haliotis discus hannai* (sea abalone) (n = 9) had been acclimated to either high or low temperature and then subjected to heat shock or no heat treatment, and mass spectrometry metabolite profiles were compared [22]. When analyzed using RVA, the CV distribution of heat-shocked, cold-acclimated abalone was significantly lower than control: control 29%, heat shock 24% (Wilcoxon t-test, *p* < 0.001) and the median CV decreased from 25 (control) to 20 (heat shock) (KS-test, d = 0.18, *p* < 0.001) (Additional file 1: Fig. S2B). High temperature-acclimated abalone groups exhibited a significant change in CV distribution profiles in response to heat shock (KS-test, d = 0.077, *p* = 0.033), representing a more narrowed distribution for the heat shock group, though the CV means were similar (Additional file 1: Fig. S2B). Together, re-analysis of the mayfly and high temperature-acclimated abalone data highlight that RVA profiles can detect even small changes reflecting intra-group metabolome variation and CV distribution changes imparted by acute stress, even after a stress acclimation period.

### Proteomics data

**Fig. 4:**
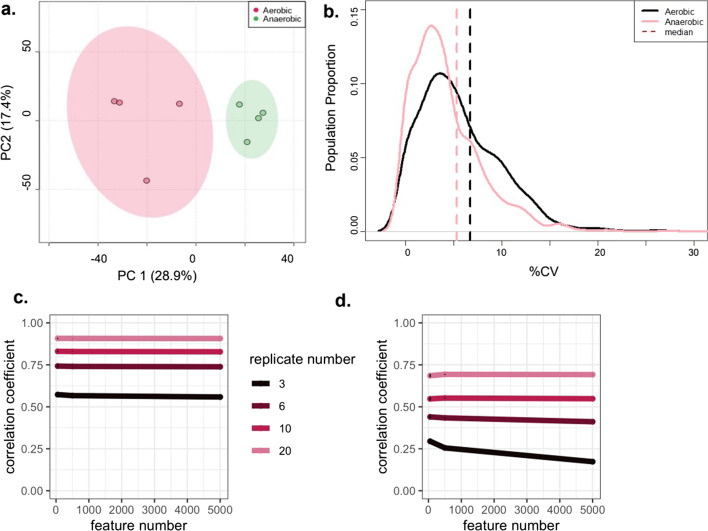
RVA of proteomics data and simulation analysis. **A** Principal component analysis of proteomic data from anaerobic and aerobic *E. coli* cultures, shown in green and red respectively. **B** CV distribution plots for anaerobic (pink) versus aerobic (black) *E. coli* cultures. **C**, **D** Simulated data with 3, 6, 10 or 20 replicates using 50, 500 or 5000 features. The standard deviation was modeled at 0.5 of the mean (**C**) and 0.23 of the mean (**D**)

We next set out to establish whether the canalization of variation following acute stress could be observed in proteomics data sets. We first looked at data from an in-house proteomic study investigating *E. coli* cell cultures grown under aerobic or anaerobic conditions (n = 4). 2D PCA score plots indicated less variation across both PC1 and PC2 in the anaerobic group (Fig. 4A), while RVA revealed a significant difference in CV distribution (K–S test, d = 0.19, *p* < 0.001) as well as a trending towards smaller CV mean (7.7% and 6.6%) and CV median (6.7% and 5.3%) for the anaerobic group (Fig. 4B). We followed this analysis by mining the Pride proteome archive database [23] to search for additional external examples, including an investigation of 48-h PEG-induced drought stress on *Triticum aestivum* L (bread wheat) (n = 3) [24]. Our RVA analysis demonstrated reduced CV distributions in drought-stressed proteome profiles (K–S test, d = 0.47, *p* < 0.001), with changes in mean and median CV values in the control group (mean = 42%, median = 29.8%) as compared to the stressed group (mean = 25%, median = 11.1%, Wilcoxon t-test, *p* < 0.001; Additional file 1: Fig. S3). Thus, both prokaryotic and eukaryotic proteome datasets provide evidence that a reduction in intra-group variation in response to acute stress applies to diverse classes of omics data.

We also tested a second proteomics data set from an in-house project. The experiment challenged *Methanocaccous voltae* to grow on different sources of iron and sulfur [25]. A comparison of *M. voltae* grown on the canonical source of iron and sulfide (Fe(II)/HS^−^) versus pyrite (FeS_2_) revealed a decrease in the CV of protein abundances when the cells were required to mobilize Fe directly from the mineral pyrite. The CV distributions between the FeS_2_ and the Fe(II)/HS^−^ proteomes were significantly different (KS test, d = 0.27616, *p* < 0.001) (Additional file 1: Fig. S4). The mean and median of the FeS_2_ cultures (mean = 18.5, median = 13.2) were also decreased compared to the Fe(II)/HS^−^ cells (mean = 25.8, median = 25.5) as 881 of 1242 proteins had a smaller CV (Wilcoxsons t-test < 0.001). These data supported our model of reduced variation in response to acute stress because growth on pyrite represents an energetic challenge for methanogens [25, 26].

The deep coverage and compact proteome of *M. voltae* present an opportunity to gain insight to proteins and pathways responsible for the reduced CV under stress. Pathway analysis through the lens of RVA highlighted that in the pyrite condition, proteins associated with stress response as well as iron/sulfur trafficking and storage had a decreased CV. A deeper look into stress-related proteins with a lower CV in the pyrite cultures showed they were part of biological (CRISPR) and environmental stress (heat shock/universal stress response) pathways. There were 14 stress-related proteins in the sulfide cultures, that had a lower CV compared to the pyrite condition, which are primarily involved in DNA repair and unfolded protein response. The other proteins with a lower CV in the sulfide condition were broadly involved in transcription and translation. Together, these proteomics data suggest that cellular stress response becomes more uniform through canalization of the important pathways.

### Exceptions to the model

Through mining the Metabolomics Workbench data repository, we determined that not all datasets exhibit this relationship between variation and stress. Three of the metabolomics datasets examined did not display a significant change in CV distribution between control and treatment groups. After a thorough analysis of experimental design, the exceptions were classified into two categories. The first category was for metabolomics analyses conducted using targeted rather than global approaches. This was the case in an analysis of isotopically labeled carbon in 256 specific metabolites to evaluate heat shock in *Caenorhabditis elegans* (nematode) [27]. CV distributions of the data from heat shocked and control groups were not significantly different (K–S test, d = 0.04, *p* = 0.83). Indicating that CV canalization is not universally present across metabolic pathways. An NMR metabolomics study analyzing cadmium exposure in *Danio rerio* (zebrafish) embryos did not show a difference in CV distribution between control and treatment groups (K–S test, d = 0.27, *p* = 0.17) [28]. As with the nematode study, this was a targeted analysis in which only 33 zebrafish metabolites were measured. This raises the important point that not all metabolites or pathways will show canalization. We hypothesize that targeted analyses may miss canalization because not all pathways need to display the effect in order to change the overall CV of a given class of biomolecules.

Other exceptions to the stress-induced CV profile changes involved chronic rather than acute stress. A blood plasma metabolomics study of Chronic Fatigue Syndrome (CFS) in both male (control = 18, CFS = 22) and female human (control = 23, CFS = 21) patients revealed that the CV distribution significantly increased in patients suffering from chronic fatigue compared to healthy control subjects (males: KS-test, d = 0.10, *p* < 0.001; females: KS-test, d = 0.10, *p* < 0.001), and the mean values increased slightly as well (males: 32% to 34%; females: 36% to 39%). Given our observations that the period of stress and/or recovery time impacts CV distribution, it is possible that in contrast to acute stress, chronic stress may result in an opposite trend and a corresponding increase in CV distribution patterns.

### Simulations and mean–variance relationships

The fact that targeted or less than global data failed to display canalization was worth further investigation. NMR datasets typically report on tens to hundreds of metabolites, while mass spectrometry-based proteomics and metabolomics data set usually contain a thousand or more spectral features. We hypothesized that the number of features comprising the CV distribution may affect statistical power to discern differences between data sets. To test the impact of data characteristics on RVA, authentic CV profiles were simulated by varying feature number (50, 500, and 5000), replicate number (3, 6, 10, and 20), and the ratio of feature mean to standard deviation. These values were selected as they are reasonable representations of different omics experimental designs. To begin, a CV profile was simulated from individual feature means and standard deviations from the mayfly data [20]. The CV profile was then randomly sampled 1000 times varying the number of features and replicates. Calculating the correlation coefficient between the “known” and sampled CV distributions revealed that more replicates in the experiment and a smaller ratio of standard deviation to the mean improves accuracy (Fig. 4C,D). Unexpectedly, the number of features is not a predictor of accuracy of CV distribution calculations as we had hypothesized. The number of biological replicates and the variance of a specific feature, however, are primary considerations. The power of RVA to detect a canalization of CVs positively correlates with the number of biological replicates.

A final test was performed to determine if the canalization of CVs due to stress could be the result of a technical artifact. The most likely source for introduction of an error is in the measurement of feature intensity, as observed in RNA-seq studies [29]. It is common for instruments to more accurately record signals for high intensity features. If this was the case, there would be a negative relationship between the variances and means of features using the data presented here. Analysis of the relationship between the mean and variance [30] in the mayfly dataset revealed the opposite trend. There was a very strong positive linear relationship between mean and variance (Additional file 1: Fig. S5) in both control and stress conditions. Therefore, we conclude that the CV is an appropriate statistic for standardizing these data for comparisons.

## Discussion

The analysis presented here identifies a correlation between variability of biological replicates and cellular stress that can be quantified in omics data. By repurposing CV as a statistic of merit, a stressed phenotype (phenome) was identified. This suggests that our RVA method can help to characterize CSR and to assess the presence and recovery from stress in biological systems.

Reduced variation in a population may be an unappreciated property of the phenome. A metabolic bottleneck or convergence (i.e. a single optimum solution to resource use) [31–33] is one possible mechanism to explain this behavior. We also propose that the change could be less of an active CSR pathway initiation and more of a passive reaction where ancillary metabolic pathways are suppressed in the perturbed organism. in this scenario, lack of nutrients or presence of negative factors (stress) on the system activates CSR and a down-regulation of other pathways to mitigate the physiological effects of stress [34]. This is consistent with studies that show trade-offs in energy allocation to alleviate competing physiological tasks during food scarcity is associated with physiological variation [35]. The proteomics data presented here for *M. voltae* grown on pyrite_2_, a less bioavailable source of Fe and S [25], fits the convergence model. RVA reinforced the idea that cells grown on pyrite were under stress, because the CV profile was significantly smaller than cells grown on iron and sulfide. In this case, *M. voltae* appeared to access classic stress management pathways including heat shock response, unfolded protein response and universal stress response [36]. Importantly, proteins from all of these categories had smaller CVs in the stressed group.

The mechanisms that result in canalization remain unknown; however, the ability to observe and quantify a population-level response provides a valuable perspective on the phenome. Whether it is activating CSR, turning down auxiliary pathways or a combination of both, our analyses demonstrate that acute stress can lead to decreased variation in omics data. At a deeper level, changes in population CV could be due to a gradient of CSR or temporal variations in stress response at the level of individuals. Single cell analysis of *Xenopus* (clawed frog) oocytes investigated this idea, by studying activation of the MAPK cascade to progesterone [37]. Ferrel et al. determined that patterns of protein phosphorylation in the population exhibited a bimodal distribution, with individuals responding to stress not gradually, but as if a switch had been flipped [37]. Research along this line, using RVA, will help to answer an ongoing and fundamental question about CSR: does it function as a rheostat or a switch? Additionally, RVA provides a finite characterization that can help identify the physiological mediators responsible for the canalization of a stressed phenotype.

CV as a global bottom-up statistic holds much potential; however, it is not without limitations. As we have shown, not all data sets follow the trend outlined here. Commonalities of studies that did not have reduced variability in “stress” groups included the presence of chronic stress and the use of a targeted rather than nontargeted analytical approaches. Chronic stress on a system is a known cause of deleterious mutations that lead to homogeneity and can result in disease, cancer and even death [38]. Data that support a reduced CV are from systems under acute stress that did not cause overt cellular death, an immediate disease state, or permanently altered CSR. For the wild oat and cancer cell data, a temporal RVA analysis showed that dampened CSR occurs in parallel with increased CV profiles that trend towards controls. We hypothesize that stress pathways will have specific time dependencies, which could explain why a change in CV distribution is not observed in some experiments. Further investigation into temporal response and return to a non-stressed phenotype are exciting topics for future research.

The use of RVA along with the standard statistical workflow for omics adds a new dimension to the data, especially where standard models requiring homogeneity of variances are not appropriate. We believe RVA will prove to be an important metric for the rapidly expanding field of phenomics. Located at the intersection of metabolomics, proteomics, and genomics, phenomics is at the forefront of human health and agricultural research. RVA helps describe the phenome of a population and is straightforward to generate. Upon further development, RVA could potentially be used as a predictive tool to pinpoint early changes in metabolite or protein levels that are indicative of stress or future disease. RVA also has implications at the juncture of stress response and resistance. It has been shown that repeated exposure to acute stress can result in long term phenotypic changes, as observed in antibiotic-resistant *E. coli* populations, herbicide-resistant weedy species[39], and prolonged stress adaptation in *Drosophila melanogaster* [39–41]. The nuances of the relationship between intra-population variability (the variome) and stress response are a gap in knowledge and a promising area for additional study.

## Methods

For previously published data, experimental details can be found in the respective publications. The *Sus scrofa* study analyzed machine learning techniques to identify biomarkers of hemorrhagic shock. Changes in CV were noted in this paper, but not further analyzed [17]. The effect of Bio Orthogonal Non-Canonical Amino Acids on *E. coli* was evaluated at the metabolite level, analyzing the addition of either AHA, HPG or Methionine [18]. Methionine sensitive cancer cells were subjected to methionine starvation with homocysteine replacement in the media, with the metabolite changes tracked over time [19]. The next study focused on heat shock treatment on mayflies to analyze stress tolerance, using GC–MS for metabolomics analysis [20]. A mouse model used to evaluate malnutrition was the next study, analyzing MS based metabolome changes [21]. The last two examples used in the metabolomics section came from a study on heat stress in abalone, studying metabolome effects of heat stress after a high or low temperature acclimation [22]. The proteomics data set utilized here analyzed drought stress on bread wheat [24].

### Metabolomics analysis of heat shocked *Avena fatua*

*Avena fatua* plants were grown from seeds as described in Burns et al. [42]. After three weeks of growth, plants (n = 8) were placed in a temperature-controlled chamber for 24 h at 40 C. Shoots were harvested at 0, 6, 24, 48, and 100 h after heat shock, immediately placed in liquid nitrogen, and stored at − 80 °C for metabolite extraction. Frozen tissue was ground for 1 min in liquid N_2_ with a mortar and pestle. The powdered tissue (approximately 150 mg per sample) was suspended in methanol (MeOH) at 70 °C for 15 min. Samples were vortexed for 1 min and then centrifuged (25,000 g, 10 min, 4 °C) to remove cellular debris from the soluble fraction. To precipitate proteins from the soluble metabolite fraction, ice cold acetone was added at a ratio of 4:1 acetone: extract and stored at − 20 °C overnight, followed by centrifugation (25,000 g) at 4 °C for 10 min. The resulting supernatant fraction was dried and stored at − 80 °C. Prior to analyses by LC–MS, samples were resuspended in 40 μL of 50% HPLC grade water / 50% MeOH. MS-based analysis of polar metabolites was accomplished using an Agilent 1290 ultra-high performance liquid chromatography (UPLC) system coupled to an Agilent 6538 Accurate-Mass quadrupole Time of Flight (TOF) mass spectrometer, using a HILIC column (Cogent diamond hydride HILIC 2.2 µM, 120 A, 150 mm × 2.1 mm Microsolv, Leland, NC) for metabolite separation. The gradient for separation started with a hold of solvent B (0.1% formic acid in acetonitrile) for 2 min at 50%, followed by a gradient ramp of 50–100% B over fourteen minutes. Then an isocratic hold at 100% solvent B for one minute, with a return to initial conditions. Mass analysis was conducted in positive mode with a capillary voltage of 3500 V, dry gas temperature of 350 °C at a flow of 8 L/min and the nebulizer was set at 60 psi, injecting 2 µL sample volumes, with blanks run intermittently between samples. Data acquisition parameters were as follows: 50–1000 mass range at 1 Hz scan rate with a resolution of 18,000. Accuracy based on calibration standards was approximately 5 ppm.

### Proteomic analysis of *Escherichia coli* grown under aerobic or anaerobic conditions

Proteomics analysis of aerobic versus nonaerobic *E. coli* cultures was carried out on MG1655 (K12) in LB media at 37 C. Four replicate cultures were started with a 5 μL inoculation from an overnight culture and grown under an atmosphere of nitrogen or ambient air until harvest at mid-log phase (0.4 OD for the aerobic samples and 0.3 OD for anaerobic samples). Cells were pelleted using centrifugation and proteins extracted immediately. The cell pellets were resuspended in 0.1 M Tris–HCL pH 7.5 buffer with 8 M urea and subjected to three freeze/thaw cycles in liquid Nitrogen, followed by ultrasonication for 5 min (Biologix -Model 13,000). Samples were centrifuged and the resulting supernatant was removed and proteins precipitated from it using ice cold acetone and stored at – 20 C for 1 h. The precipitated proteins were centrifuged, the supernatant was removed and the protein pellet was resuspended in 0.1 M Tris–HCL pH 6.8, 5 um EDTA, 50 mM N-ethylmaleimide in 6 M urea. This sample was transferred to a 3 K MWCO Nanosep centrifuge device and a modified FASP digestion was carried out. The sample was reduced with an excess of DTT and alkylated using 50 mM Iodoacetamide. The samples were washed four times with 50 mM ammonium bicarbonate pH 7.8 and then digested using sequencing grade Trypsin at a 20:1 protein: protease ration for 18 h. Samples were run on a Dionex Ultimate 3000 Nano UHPLC equipped with an Acclaim PepMap 100 C18 trap column (100 μm × 2 cm) and an Acclaim PepMap RSLC C18 (75 μm × 50 cm, C18 2 μM 100A) for separation. Mobile phase A was 0.1% formic acid in HPLC grade water and B was 80/20 acetonitrile: water. Peptides were separated at 0.6 nL/min. using a linear solvent gradient from 3–30% B over 120 min. The LC system was coupled with a Bruker maXis Impact with captive spray ESI mass spectrometer was used for data collection of spectra from 150 to 1750 m/Z at a maximum rate of 2 Hz for precursor and fragment spectra with adaptive acquisition for highly abundant ions. Data dependent MS/MS was used to collect sequence information on the 5 most abundant ion per full scan. Data analysis was done using MaxQuant (v1.6.4.0) and Perseus (v1.6.4.10).

### Mining of public data

Data was obtained from the Metabolomics Workbench [43] and the PRIDE proteomics repository [23]. The archives were searched for data sets that matched “stress” in the keyword search. If the summary described an omics data set that evaluated a stress or perturbation and a control group, both with at least three biological replicates, the uploaded data set was evaluated. If the data provided was in a raw format (e.g. “sample.d” datafile) the set was discarded in order to avoid potential bias from our in-house processing pipeline. If the data was in a final, processed tabular format and experimental conditions were clearly described, the data was used. Reasons for not using a data set included lack of clearly defined experimental and control groups, undecipherable sample codes, or incomplete data inclusion. Data sets that met the criteria of containing stress and control groups with at least three biological replicates, were evaluated by replicate variation analysis (Additional file 2: Table S3).

### Statistical analysis

CV statistics were calculated using the standard deviation and the mean of individual metabolites or proteins in a group. The standard deviation was taken as a ratio to the mean and reported as a percentage. This was done for every detected metabolite feature or protein to obtain the distribution of the omic population. Statistical analysis was carried out in R [44] and distribution plots were made using ggplot2 [45] and ggridges [46], PCA plots, histograms of CV, distribution plots, and distribution statistics of mean and median were all calculated and plotted. A two sample Kolmogorov–Smirnoff (KS) test was utilized to analyze for the empirical distribution functions of the control and the treatment groups. The two sample KS test describes the differences between shape and location of the two distributions being tested using the d statistic with a calculated *p* value. A larger d statistic indicates a larger change between the two distributions being compared [16].

### Simulated data analysis

The process of simulating these CV distributions requires two levels of simulations—first, a simulation of the population level CV distribution and second, simulations of the individual replicates sampled from these CV distributions. Therefore, the “true” CV distributions across the population level were simulated first. For this, both the means and standard deviations were simulated for each omics feature. The Mayfly treatment dataset presented in Fig. 2 was used to parameterize simulations. The means were drawn from a normal distribution with a (1) mean equal to the log(mean) of the Mayfly dataset to disallow negative values and (2) a standard deviation equal to the standard deviation of the log(mean) of the dataset. Each mean also required a corresponding simulated standard deviation. Within the Mayfly treatment dataset, the standard deviation varies from 0.02 to 1.65x of its corresponding mean, with a mean standard deviation fold-change of 0.23. Therefore, we tested both 0.23-fold and 0.5-fold of the mean and 0.1 as the standard deviation to randomly assign each mean a corresponding standard deviation. Finally, the CV was calculated for each mean-standard deviation pair to create the “true” CV distribution. Forty distributions were simulated.

Random sampling from each of the CV distributions was simulated as follows: For each mean and standard deviation pair, varied numbers of replicates were drawn, and the CV was computed. The Spearman’s correlation between the CV for these simulated samples and the “true” CV simulated in the first step was determined. The process was repeated 1000 times for each replicate and feature number combination.

## Supplementary Information

**Additional file 1. Figure S1.** **a** Distribution plots of CV of NMR metabolite feature profiles for the non-canonical amino acid treated cultures of *E. coli*. **b** CV profiles of metabolites in methionine dependent cancer cells with methionine (MET) or homocysteine (Hcy). **c** CV profiles of metabolites from replicates of mayflies that were exposed to heat stress (pink) and the analogous control group (black). **Figure S2.** Undernourished mouse model studies and Temperature Acclimated Abalone. **a** Distribution plots of CV metabolite features from control mice (black) and malnourished mice (pink). **b** Distribution plots of CV metabolite features from replicates of cold (left panel) or high temperature (right panel) acclimated *Haliotis discus hannai* that were exposed to heat stress (pink) and the analogous control group (black). **Figure S3.** Distribution profile plots of proteomic data collected on wheatleaf (black) and wheatleaf that has been exposed to drought stress using PEG (pink). **Figure S4.** Distribution profile plots of proteomic data collected on *M. voltae* grown under canonical sulfide (Fe(II)/HS^−^) (black) and *M. voltae* that has been exposed to mineral stress through growth on pyrite (FeS_2_) (pink). Mean from 25.8 to 18.5% Median (shown) from 21.5 to 13.2%. **Figure S5.** Relationship between feature mean and variance for control replicates from the mayfly data (**a**) and the heat stressed mayfly replicates (**b**).

**Additional file 2. Table S1. **Protein CVs.** Table S2. **Data set details.** Table S3. **Metabolomics workbench search.

## Data Availability

The datasets generated and/or analysed during the current study are available in the Metabolomics Workbench (https://www.metabolomicsworkbench.org/) and PRIDE (https://www.ebi.ac.uk/pride/) repositories or available on request.
