# Supplementary material for: Acute stress reduces population-level metabolic and proteomic variation
Source: BMC Bioinformatics. 2023 Mar 7;24:87. doi: 10.1186/s12859-023-05185-4 (PMC9993721; doi:10.1186/s12859-023-05185-4)
Supplement: Supplementary file 1 — Additional file 1. Figure S1. a Distribution plots of CV of NMR metabolite feature profiles for the non-canonical amino acid treated cultures of E. coli. b CV profiles of metabolites in methionine dependent cancer cells with methionine (MET) or homocysteine (Hcy). c CV profiles of metabolites from replicates of mayflies that were exposed to heat stress (pink) and the analogous control group (black). Figure S2. Undernourished mouse model studies and Temperature Acclimated Abalone. a Distribution plots of CV metabolite features from control mice (black) and malnourished mice (pink). b Distribution plots of CV metabolite features from replicates of cold (left panel) or high temperature (right panel) acclimated Haliotis discus hannai that were exposed to heat stress (pink) and the analogous control group (black). Figure S3. Distribution profile plots of proteomic data collected on wheatleaf (black) and wheatleaf that has been exposed to drought stress using PEG (pink). Figure S4. Distribution profile plots of proteomic data collected on M. voltae grown under canonical sulfide (Fe(II)/HS−) (black) and M. voltae that has been exposed to mineral stress through growth on pyrite (FeS2) (pink). Mean from 25.8 to 18.5% Median (shown) from 21.5 to 13.2%. Figure S5. Relationship between feature mean and variance for control replicates from the mayfly data (a) and the heat stressed mayfly replicates (b). [file 12859_2023_5185_MOESM1_ESM.pdf]

Figure S1

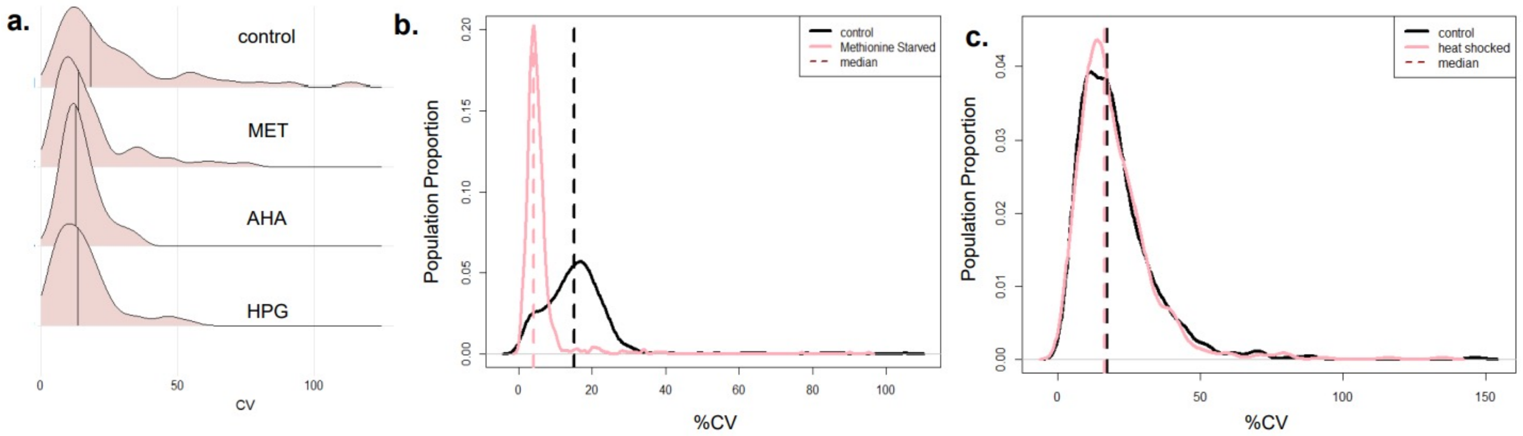

Supplementary Figure 1. a. Distribution plots of CV of NMR metabolite feature profiles for the non-canonical amino acid treated cultures of *E. coli.* **b**. CV profiles of metabolites in methionine dependent cancer cells with methionine (MET) or homocysteine (Hcy). **c.** CV profiles of metabolites from replicates of mayflies that were exposed to heat stress (pink) and the analogous control group (black).

Figure S2

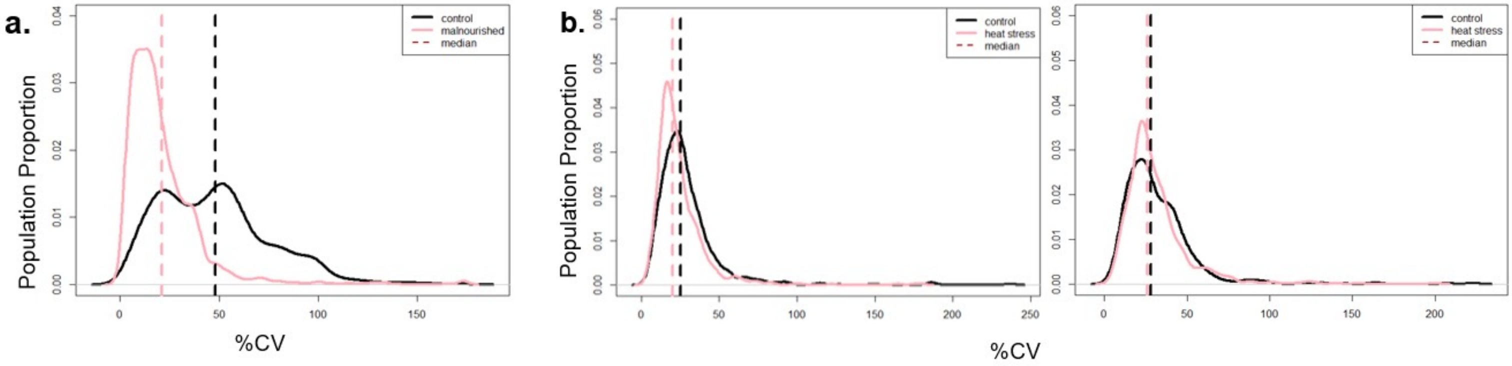

Supplementary Figure 2. Undernourished mouse model studies and Temperature Acclimated Abalone. **a.** Distribution plots of CV metabolite features from control mice (black) and malnourished mice (pink). **b.** Distribution plots of CV metabolite features from replicates of cold (left panel) or high temperature (right panel) acclimated *Haliotis discus hannai* that were exposed to heat stress (pink) and the analogous control group (black).

Figure S3

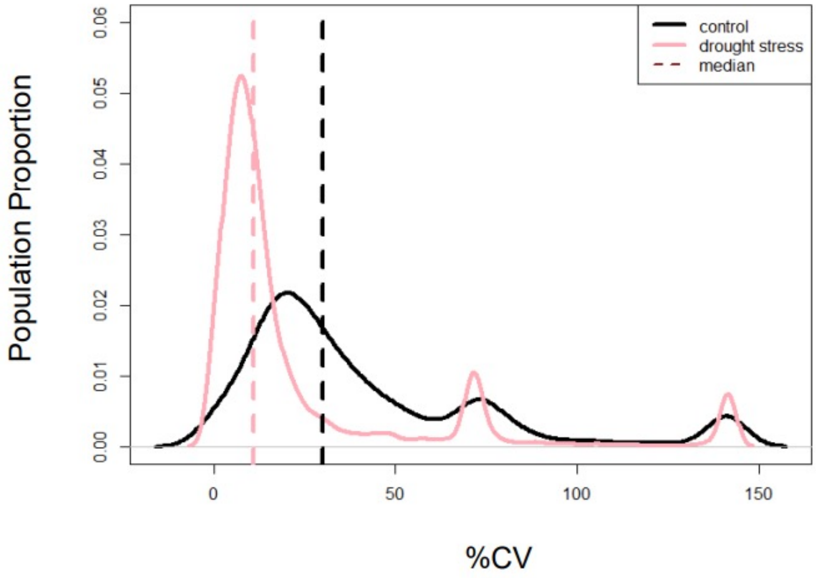

Supplemental Figure 3. Distribution profile plots of proteomic data collected on wheatleaf (black) and wheatleaf that has been exposed to drought stress using PEG (pink).

Figure S4

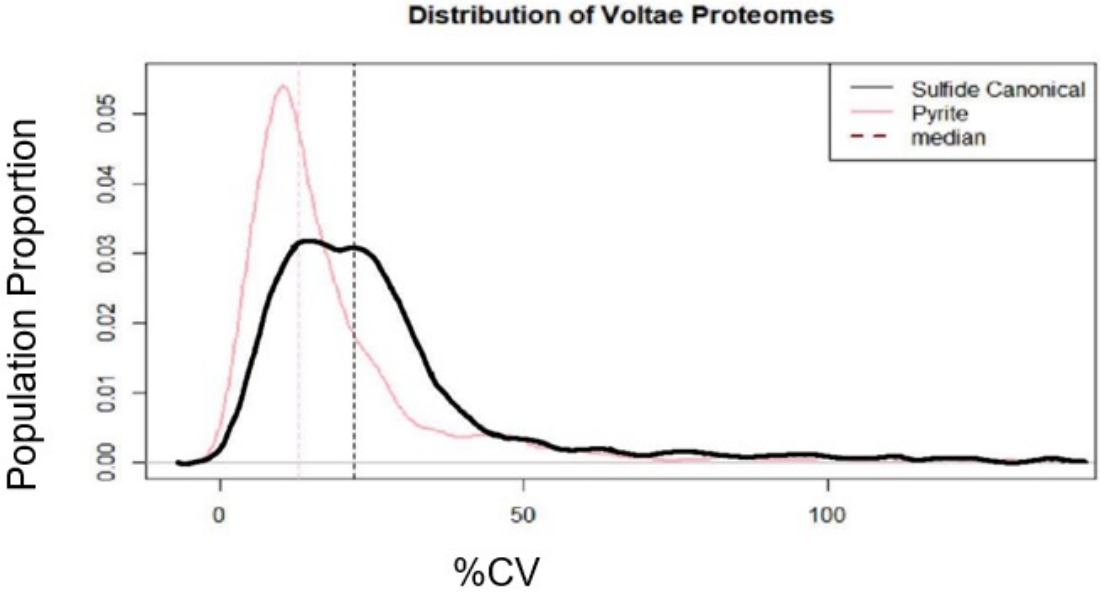

Supplemental Figure 4. Distribution profile plots of proteomic data collected on M. voltae grown under canonical sulfide (Fe(II)/HS^{-}) (black) and M. voltae that has been exposed to mineral stress through growth on pyrite (FeS_2)(pink). Mean from 25.8% to 18.5%
Median (shown) from 21.5% to 13.2%

Figure S5

a.

b.

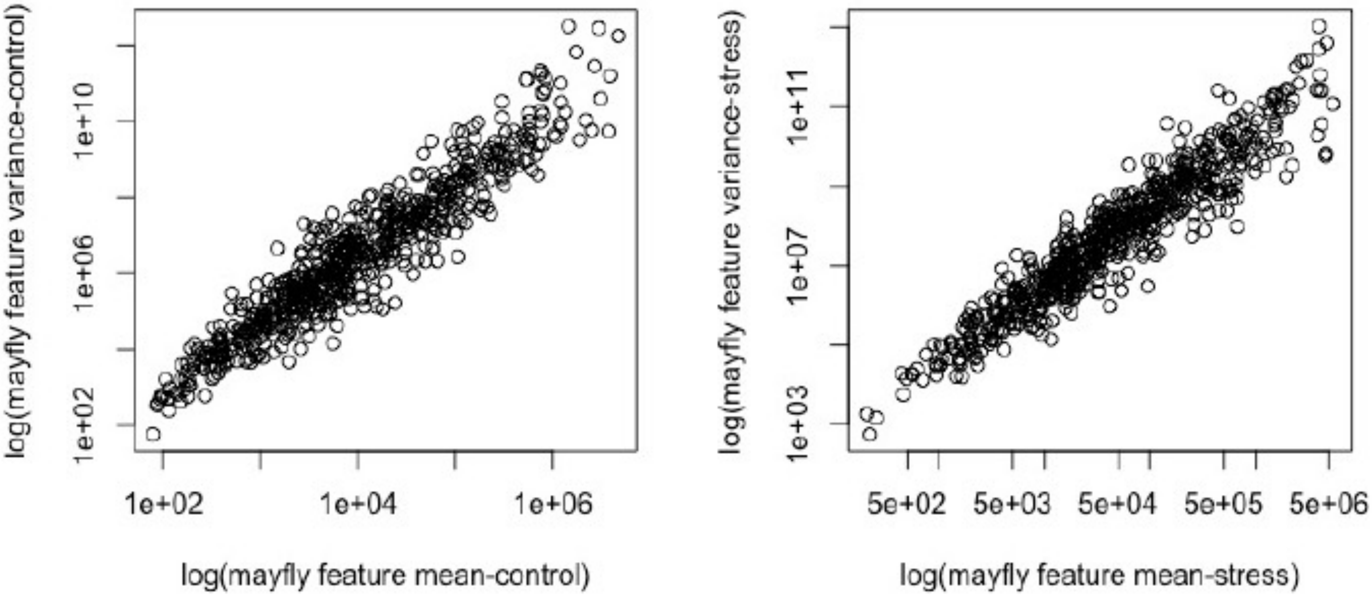

Supplemental Figure 5. Relationship between feature mean and variance for control replicates from the mayfly data (a) and the heat stressed mayfly replicates (b).
